# Flexor Hallucis Longus Transfer With Concurrent Gastrocnemius Augmentation in Neglected Tendoachilles Tears: A Case Series

**DOI:** 10.7759/cureus.65170

**Published:** 2024-07-23

**Authors:** Vijayanand B, Kannan KC, Karthikeyan S

**Affiliations:** 1 Orthopaedic Surgery, Sri Ramaswamy Memorial (SRM) Institute of Science and Technology, Chennai, IND; 2 Orthopaedics, Sri Ramaswamy Memorial (SRM) Institute of Science and Technology, Chennai, IND; 3 Orthopaedic Surgery, Sri Ramaswamy Memorial (SRM) Medical College Hospital and Research Centre, Chennai, IND

**Keywords:** surgical intervention, vas score, aofas score, gastrocnemius augmentation, neglected insertional rupture, flexor hallucis longus (fhl) transfer, achilles tendon

## Abstract

The Achilles tendon, the body's largest tendon, is often vulnerable to rupture, primarily as a result of sudden dorsiflexion of a plantar-flexed foot. This injury predominantly affects individuals in their youth and middle age. In this case series, we describe three middle-aged men with neglected insertional Achilles tendon ruptures, each presenting an average 10 cm defect. They underwent a surgical procedure involving flexor hallucis longus (FHL) tendon transfer with concurrent gastrocnemius augmentation. The FHL tendon was repositioned proximally and securely tenodesed to the proximal stump of the excised Achilles tendon. Following this intervention, substantial clinical improvements were observed at the six-month follow-up, with the American Orthopaedic Foot and Ankle Society (AOFAS) score improving from 35 to 85 and the Visual Analog Scale (VAS) pain score decreasing from 8 to 2. These results highlight the efficacy of flexor hallucis longus tendon transfer with gastrocnemius augmentation as a superior treatment option for neglected insertional achilles tendon tears characterized by substantial defects, promising improved functional outcomes and pain relief.

## Introduction

Techniques such as angioplasty or tendon transfers can effectively manage chronic Achilles tendon ruptures. Kuwada et al. classified chronic tendo-Achilles tears into four types based on the defect size: type 1 with less than 50% tear recommended treatment is cast immobilisation for eight weeks; type 2 where the defect is less than 3 cm simple end-to-end anastomosis is recommended; type 3 where the defect is 3-6 cm end-to-end anastomosis with autogenous or synthetic graft is recommended; type 4 where the defect is greater than 6 cm (chronic cases) gastrocnemius recession/free tendon grafts/tendon transfer is recommended [1]. The transfer of the flexor hallucis longus (FHL) tendon for chronic Achilles tendon conditions has been shown to not only alleviate pain but also enhance overall function [2,3]. Following flexor hallucis longus tendon transfer, the maximum plantar flexion strength is reduced by 16% to 35% when compared to the native Achilles tendon [4]. Patients also tend to experience diminished performance in single- and two-leg heel-rise tests. However, there is a lack of research that investigates patients' return to work as well as their ability to walk or engage in activities such as jumping. In the following article, we present three cases of chronic tendo-Achilles rupture managed by flexor hallucis longus transfer with tendo-Achilles augmentation.

## Case presentation

Case 1

**Figure 1:**
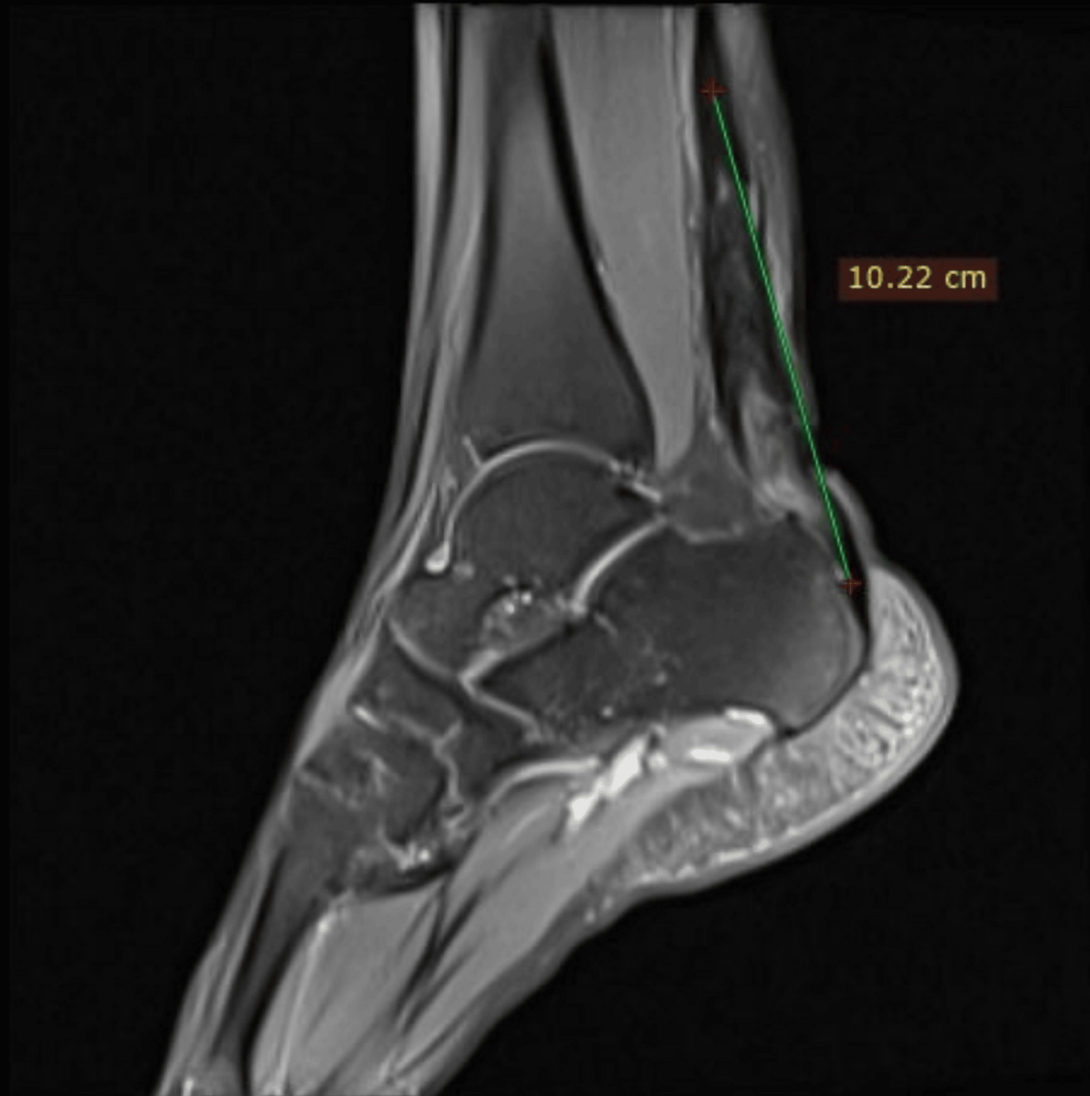
Pre-operative MRI of the ankle showing torn and retracted tendo-Achilles with gap of 10.22 cm Case 1

**Figure 2:**
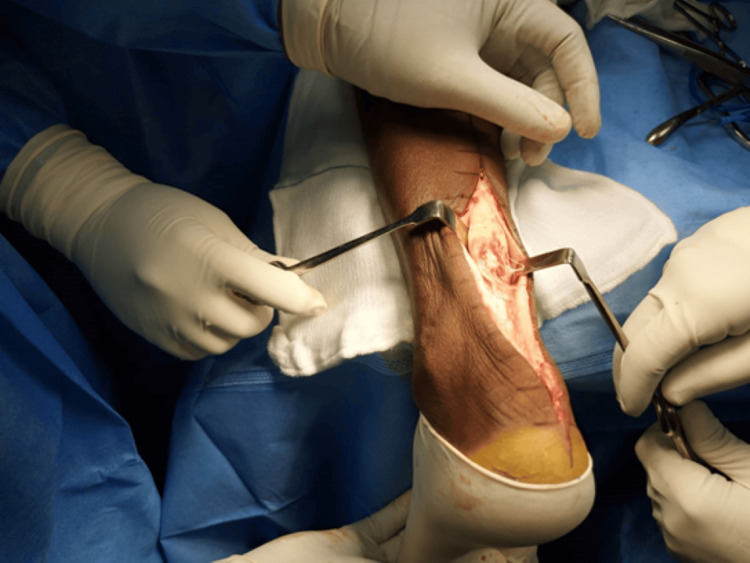
After posterior midline incision and opening of the paratenon, the torn stumps of the retracted tendo-Achilles is seen Case 1

**Figure 3:**
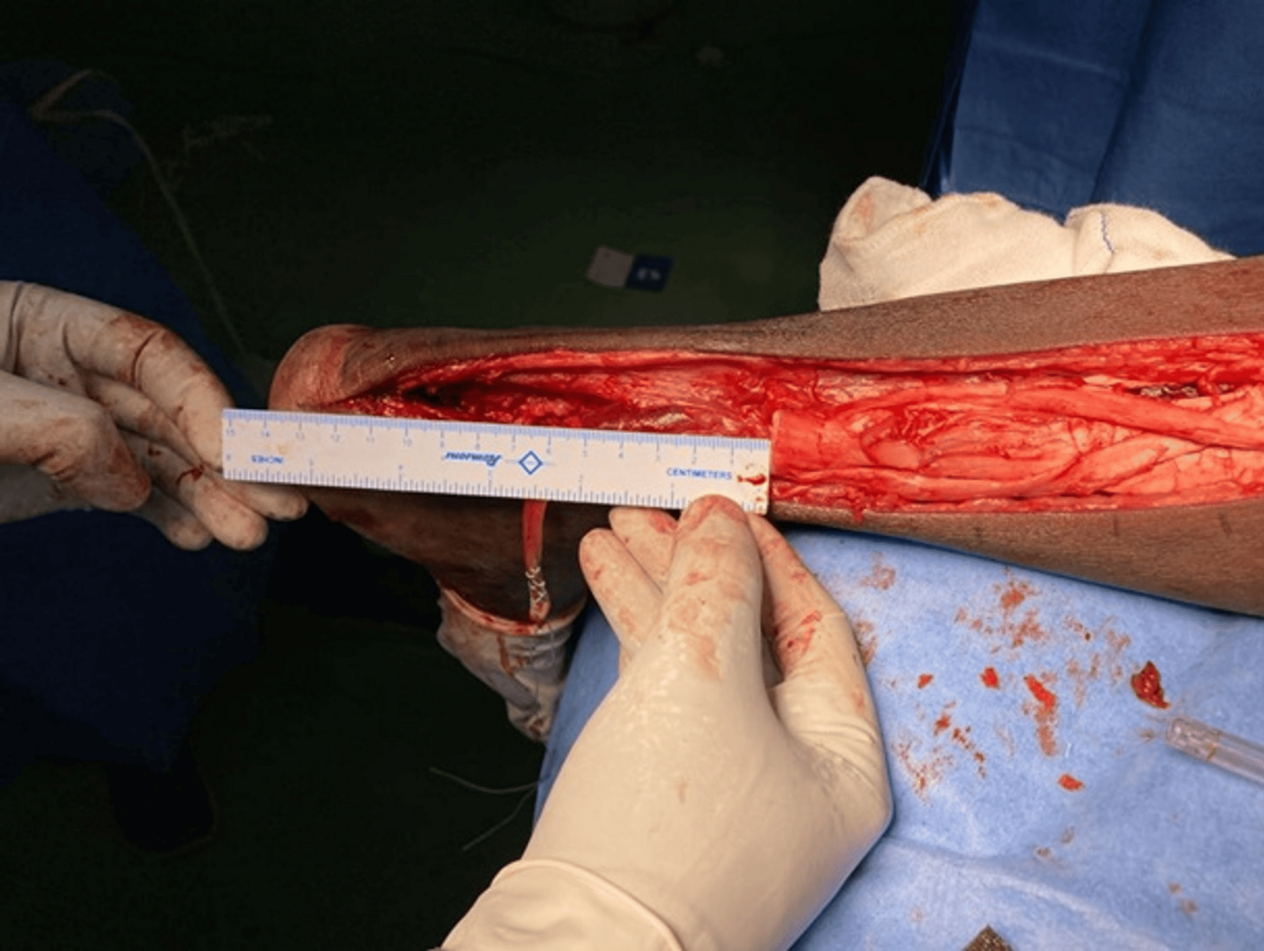
Post debridement of the torn stumps showing a defect of 12 cm Case 1

**Figure 4:**
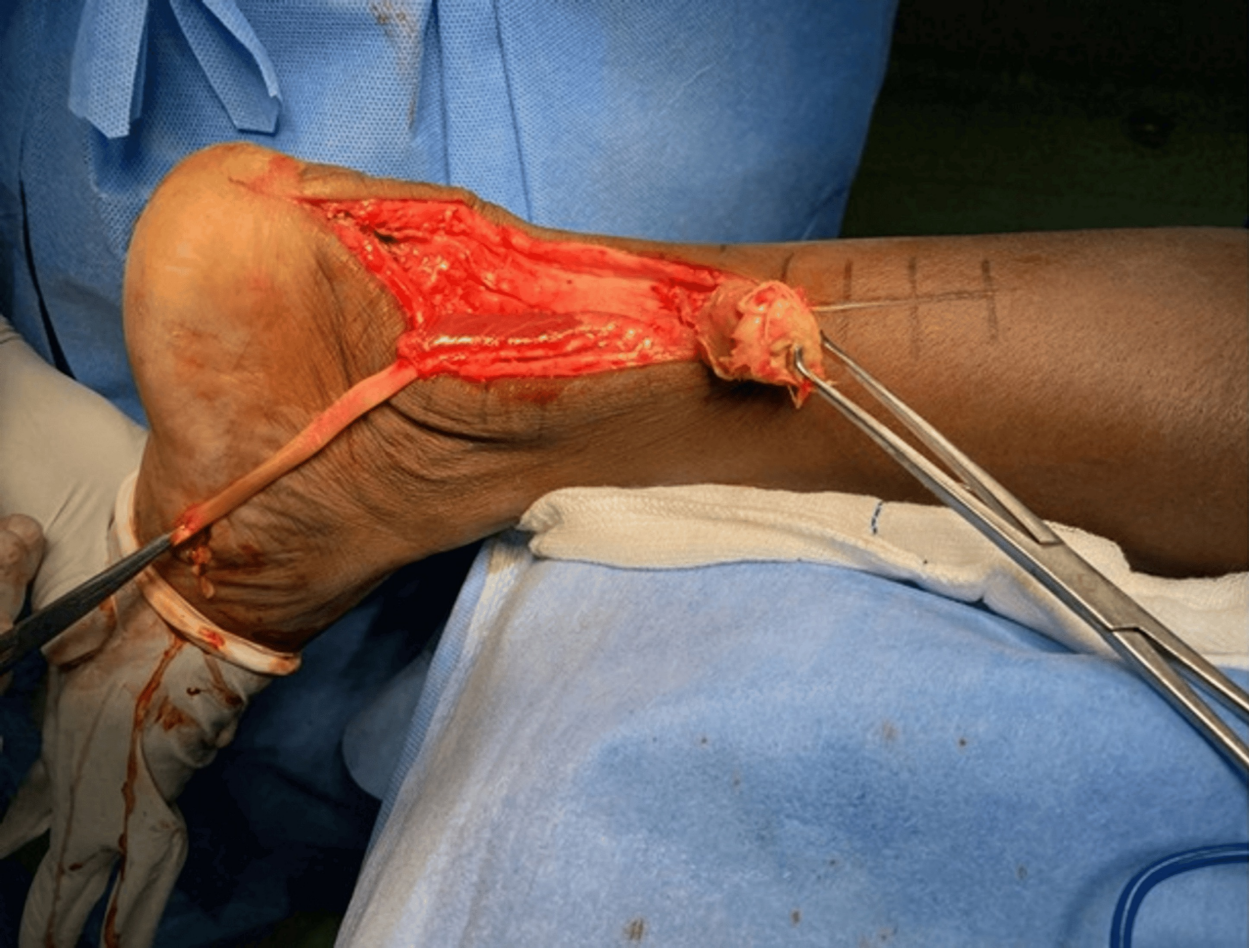
The flexor hallucis longus is harvested from the master knot of Henry and prepared for transfer Case 1

**Figure 5:**
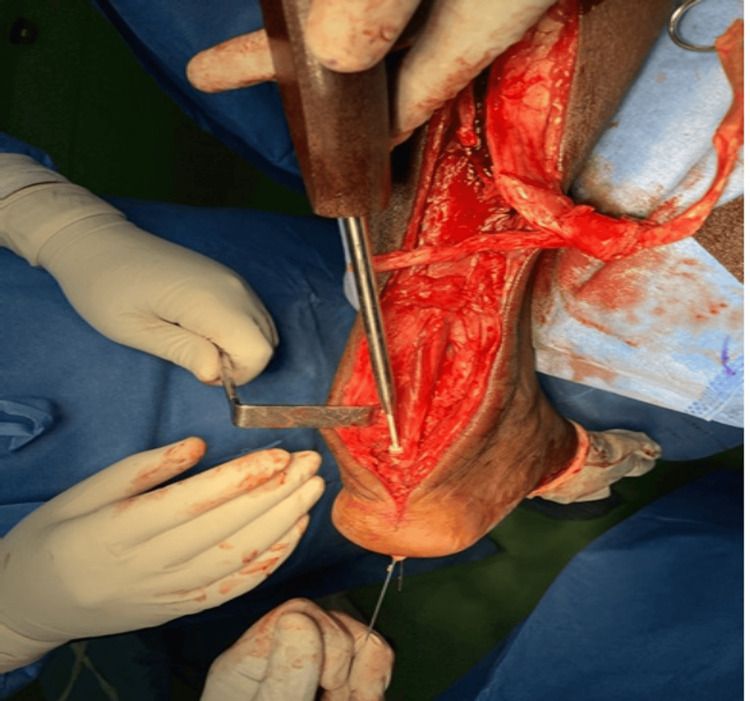
Transosseous transfer of flexor hallucis longus onto the posterosuperior aspect of calcaneum using interference screw Case 1

**Figure 6:**
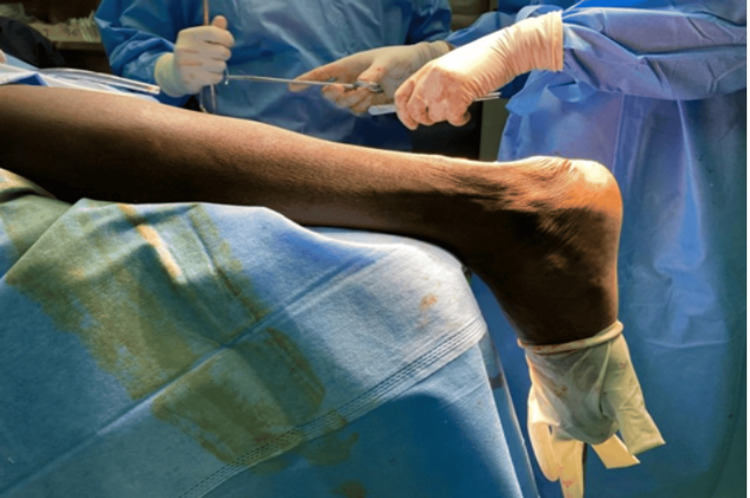
Post transfer of flexor hallucis longus, the ankle is maintained in 20° of plantar flexion Case 1

**Figure 7:**
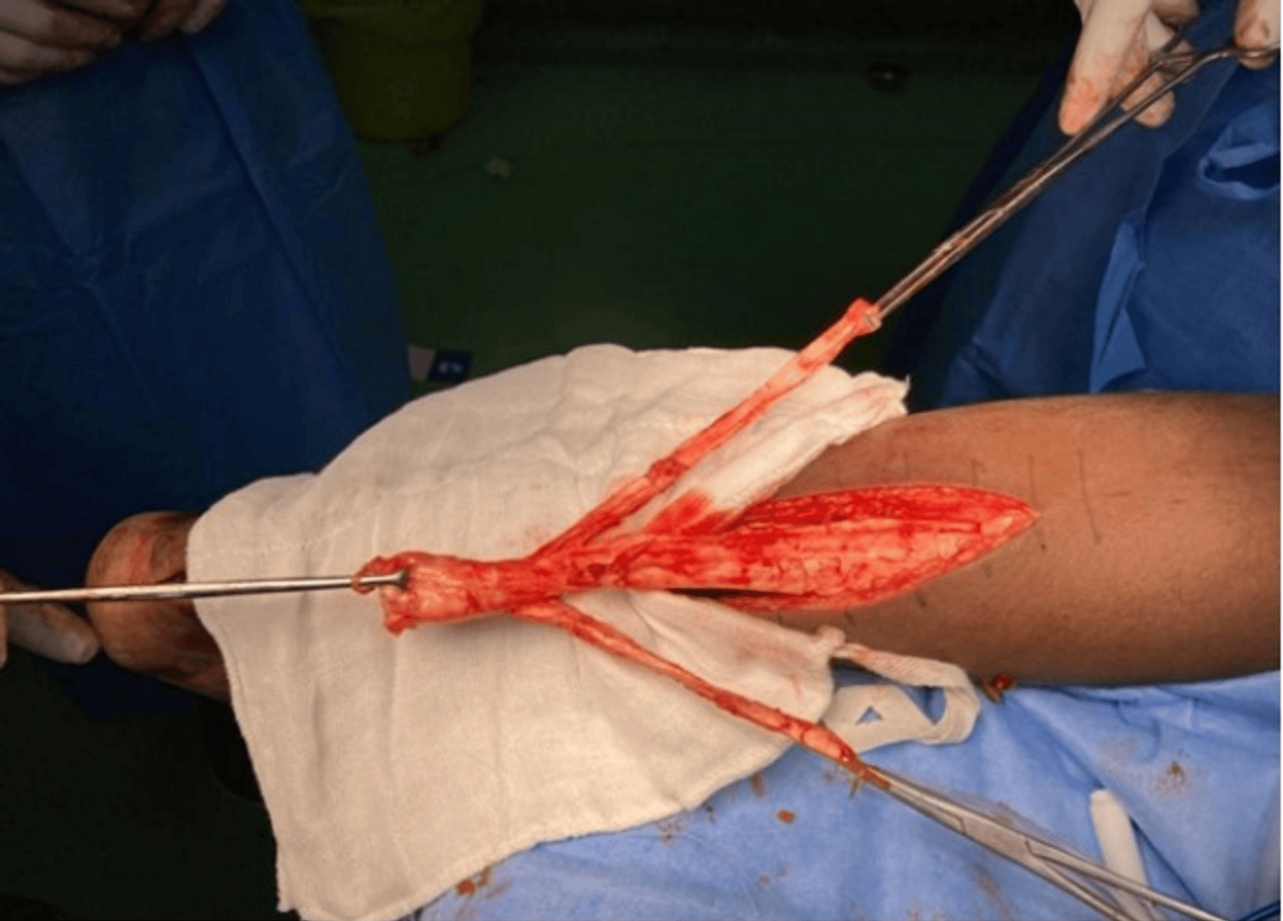
Gastrocnemius aponeurotic flaps taken on either side for turn down procedure Case 1

**Figure 8:**
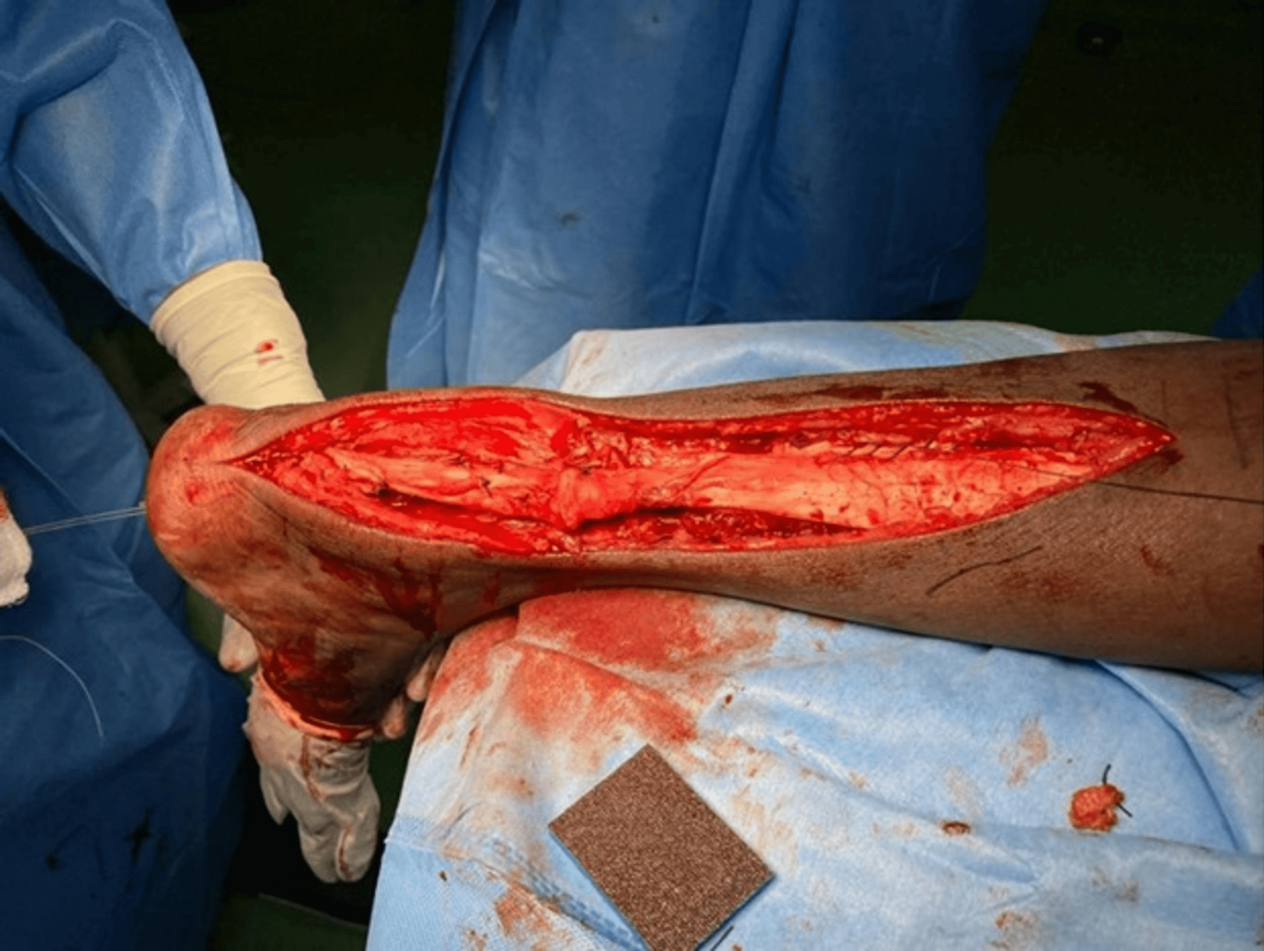
Completed flexor hallucis longus transfer with tendo-Achilles augmentation Case 1

**Figure 9:**
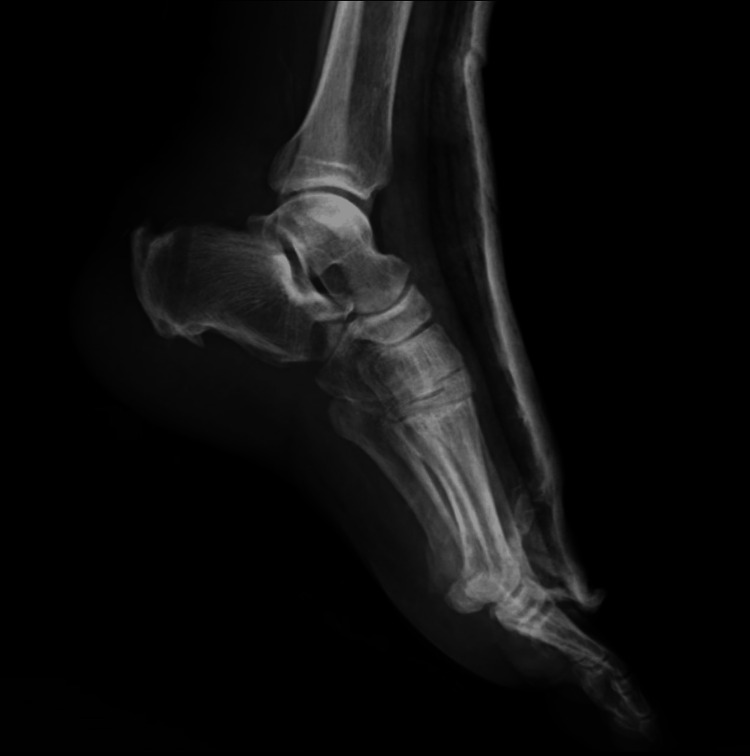
Post-operative X-ray following flexor hallucis longus transfer with tendo-Achilles augmentation Case 1

A 51-year-old male manual labourer presented with a history of difficulty walking following an injury to the back of his right heel four months ago. The Thompson squeeze test and single-leg heel raise test were positive. MRI showed chronic rupture of the tendo Achilles with tendonitis with a gap of 10.22 cm and Haglund deformity (Figure 1). The patient underwent flexor hallucis longus transfer and tendo-Achilles augmentation as he was categorised under the type 4 Kuwada classification. The patient was put in a prone position, with his ankle and foot free over the edge of the table, allowing a full range of movement. A posterior midline longitudinal incision was made, and the paratenon was opened. The retracted tendo Achilles was identified and debrided (Figure 2). Post-debridement of the edges revealed a gap of 12 cm (Figure 3). Flexor hallucis longus was identified and harvested from Henry's master knot (Figure 4). The tendon was prepared and sized to be 5 mm. A transosseous tunnel of diameter 6 mm was made 1 cm anterior to the tendo-Achilles insertion site in the calcaneum from the superior to inferior direction. The prepared graft was pulled through the tunnel and fixed with a 6 mm interference screw (Figure 5) at 20° of plantar flexion (Figure 6). Then augmentation was done using flaps taken along the aponeurosis of the tendo Achilles and turned down by 180° (Figure 7), which was sutured to the transferred flexor hallucis longus (Figure 8). After thorough lavage, the paratenon was closed, followed by skin closure in layers. Figure 9 shows the immediate post-op X-ray. An above-knee cast was applied, which was revised with increasing angles of dorsiflexion as tolerated every two weeks. Ankle ROM and partial weight bearing were allowed by six weeks and full weight bearing by 12 weeks. The preop American Orthopaedic Foot and Ankle Society (AOFAS) score was 68. Six-month post-operative and one-year post-operative AOFAS scores were 89 and 99, respectively.

Case 2

**Figure 10:**
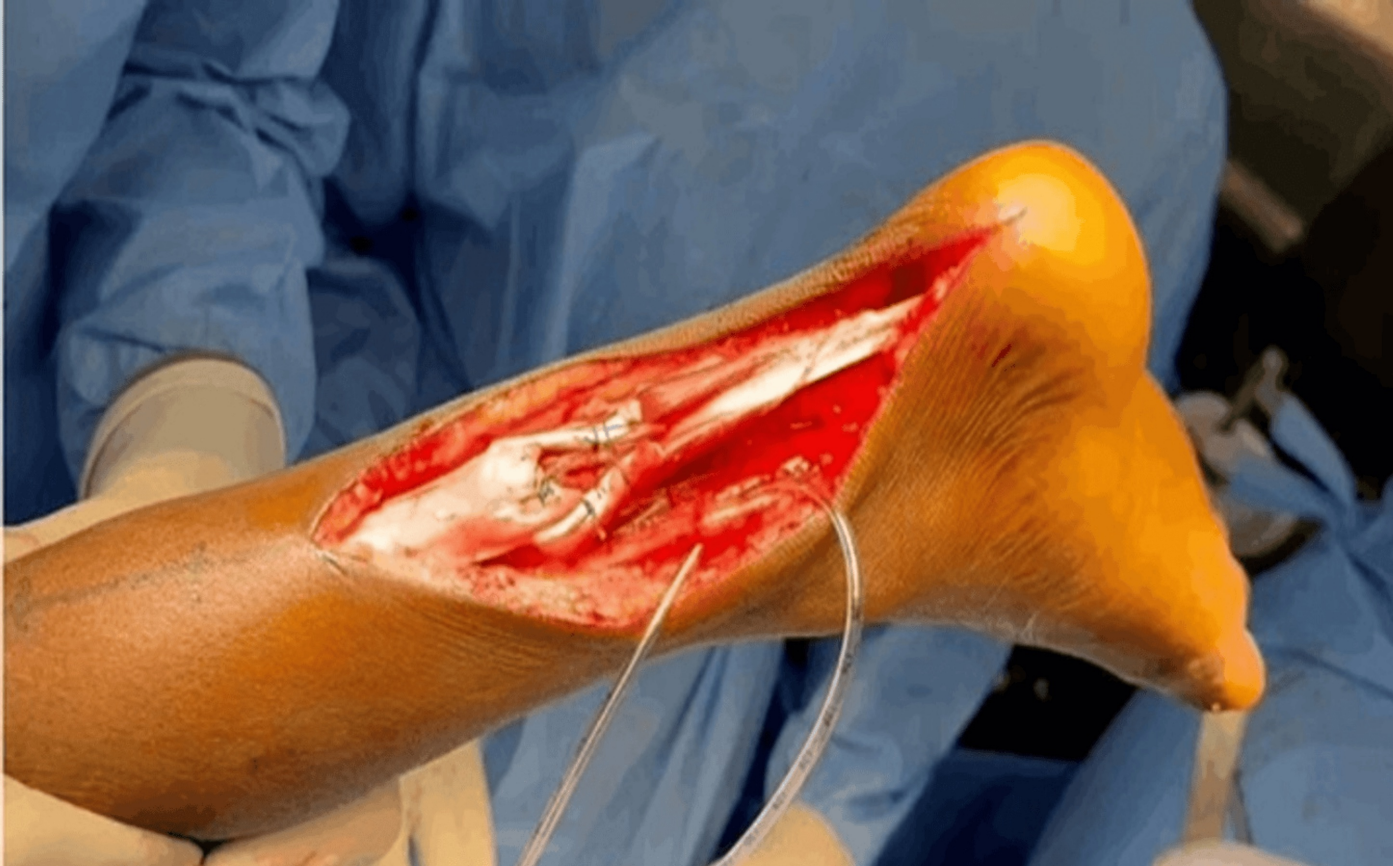
Intraoperative image showing the completed flexor hallucis longus transfer with tendo-Achilles augmentation Case 2

A 48-year-old male farmer by occupation with a history of laceration to the back of his heel on the left side that was sutured at a local hospital presented to us after three months of difficulty walking. On examination, the patient had a scar over the posterior aspect of the ankle with a palpable defect over the tendo-Achilles insertion region. The single-leg heel raise and Thompson test were positive. An MRI was done, which showed a chronic retracted tendo-Achilles tear with a gap of 12 cm. Flexor hallucis longus tendon transfer with tendo-Achilles augmentation was done using the same procedure as described in case 1, since the post-debridement gap of the tendo Achilles was more than 6 cm by Kuwada classification. The subsequent treatment course involved placing the patient in an above-knee cast with the foot in a plantar flexed position for four to six weeks, with gradual adjustments until 20° ankle dorsiflexion is achieved. After six weeks, the patient was permitted partial-weight-bearing ambulation with crutches and an ankle-foot boot. By 12 weeks, the patient had started full-weight-bearing walking. Figure 10 shows the completed flexor hallucis longus transfer with tendo-Achilles augmentation. The preop AOFAS score was 76. Six-month post-operative and one-year post-operative AOFAS scores were 85 and 100, respectively.

Case 3

A 41-year-old male machine operator presented with an inability to do a single-leg heel raise on his left side after a direct hit to his heel four months ago. An MRI showed a chronic retracted tendo-Achilles avulsion with a gap of 12 cm. Flexor hallucis longus tendon transfer with tendo-Achilles augmentation was done following similar surgical steps and post-operative protocol. Full-weight bearing was started after three months. The preop AOFAS score was 81. Six-month post-operative and one-year post-operative AOFAS scores were 90 and 98, respectively.

Results

All three patients were followed up for one year. The data of the three patients with chronic retracted tendo-Achilles rupture treated by flexor hallucis longus transfer and tendo-Achilles augmentation is tabulated below (Table 1). All the patients were classified as type IV by the Kuwada classification as the gap was more than 6 cm.

**Table 1:** Demographic data of the patients included in the study

P. No	Gender	Age	Occupation	Side	Pre-op gap as per MRI	Post-debridement gap
1.	Male	51	Manual labourer	Right	10.22 cm	12 cm
2.	Male	48	Farmer	Left	12.05 cm	13.5 cm
3.	Male	41	Machine operator	Left	12 cm	13 cm

All three patients were put on above-knee casts with 20° of plantar flexion in the post-operative period. The cast was gradually altered once every two weeks to obtain a final dorsiflexion of 20° by six to eight weeks. Partial weight bearing, as tolerated, was started by six weeks and progressed to full weight bearing by 12 weeks. By six months, all the patients were able to do single-leg heel raises without support. The power of the flexor hallucis longus was reduced when compared to the preoperative state, but it did not interfere with the patient’s gait postoperatively in all three cases. At six months and one year, the AOFAS score was calculated, which is recorded in the below table (Table 2).

**Table 2:** American Orthopaedic Foot and Ankle Society (AOFAS) score for the patients included in the study

Sl. No	Chronicity of tear	Pre-op AOFAS score	6 month post-operative AOFAS score	1 year follow-up AOFAS score	Total duration of follow-up
1.	4 months	68	89	99	12 months
2.	3 months	76	85	100	15 months
3.	4 months	81	90	98	15 months

The results show that this procedure gives better functional outcomes to the patients with regard to the ankle and foot scores. Also, all three patients in our study returned to their occupation by six to nine months post-surgery. One patient developed a superficial infection of the suture site, which was treated with regular dressings and oral antibiotics. The other two patients did not have any post-operative complications.

## Discussion

Addressing chronic Achilles tendon ruptures presents a formidable challenge for orthopaedic surgeons [5]. These chronic ruptures, distinguished by their distinct pathophysiological characteristics from acute cases, often entail the formation of significant gaps within the tendon. When these gaps are bridged by scar tissue, it can lead to functional impairments in the ankle, manifesting as ankle weakness and gait disturbances [6]. This pathophysiological complexity may be further exacerbated by the infiltration of substantial amounts of fat into the tendon gap.

Chronic Achilles tendon ruptures are prone to misdiagnosis, and up to 20% of cases may progress to a neglected state [4]. Following an Achilles tendon rupture, patients frequently experience a notable reduction in plantar flexion strength, which leads to an altered gait pattern. A modified Thompson test can be used for the clinical evaluation of tendo-Achilles tears. The test exhibited a higher rate of positive results when conducted with the patient in a prone position, with the knee flexed at 90°, as opposed to when the knee was extended. However, the inability to perform a single-leg heel rise represents a pivotal clinical sign, often necessitating the consideration of reconstruction surgery. Surgical reconstruction, as highlighted in our study, holds the potential to fully restore Achilles tendon strength, leading to marked improvements in patient mobility. However, it is imperative to acknowledge that the procedures involved in the reconstruction and augmentation of chronic Achilles tendon ruptures are challenging, and the selection of surgical techniques should be influenced by the size of the defect [1].

In our study, we observed a notable contrast with previous reports. It has been reported that between 50% and 86% of patients were able to perform a single-limb heel raise test on the operated side after the transfer of the flexor hallucis longus tendon, with comparatively poorer outcomes in cases of chronic ruptures [7,8]. In our study, a complete improvement in single-limb heel raise capability was observed after flexor hallucis longus transfer, indicating more favourable outcomes even in the context of chronic Achilles tendon ruptures. In the study by Yeoman et al. [9], they successfully managed 11 patients with chronic Achilles tendon ruptures by employing the Flexor Hallucis Longus technique in conjunction with interference screw fixation, achieving consistent and favourable outcomes with minimal complications. Furthermore, Oksanen et al. [4] reported a notable hypertrophy of approximately 52% in the transferred Flexor Hallucis Longus muscle, as observed through MRI evaluation following a chronic tendo-Achilles tear.

Interestingly, a comparable phenomenon was also observed in our current study. This observation suggests that the flexor hallucis longus tendon possesses a remarkable capacity for adaptation, which could be instrumental in achieving positive results in the context of Achilles tendon rupture management. Furthermore, Tay et al. [10] and Peterson et al. [11] reported that chronic Achilles tendon ruptures treated with two turndown flaps and flexor hallucis longus augmentation yielded satisfactory results during a two-year follow-up when addressing central defects of approximately 12 cm. In our study, we observed improvements in the AOFAS score within 12 months, indicating a satisfactory overall improvement in patient outcomes.

These findings emphasise the complexity and variability of managing chronic Achilles tendon ruptures. The choice of diagnostic tools, surgical techniques, and post-operative assessments can significantly impact patient outcomes and highlight the need for further research and evaluation in this challenging clinical area.

## Conclusions

In conclusion, our study demonstrates that the reconstructive technique using flexor hallucis longus transfer with concurrent gastrocnemius augmentation can yield good clinical and functional outcomes. We have found this surgical technique to be highly valuable in addressing the challenging cases of neglected Achilles tendon ruptures. These results underscore the effectiveness of flexor hallucis longus tendon transfer combined with gastrocnemius augmentation as a viable treatment option, but a large sample size will be required to further evaluate and validate the same, especially for neglected insertional Achilles tendon tears that present substantial defects. This approach holds the promise of not only enhancing functional outcomes but also providing significant improvement in gait patterns for affected patients.
